# Does Regional Anesthesia Improve Recovery After vNOTES Hysterectomy? A Comparative Observational Study

**DOI:** 10.3390/medicina62010154

**Published:** 2026-01-13

**Authors:** Kevser Arkan, Kubra Cakar Yilmaz, Ali Deniz Erkmen, Sedat Akgol, Gul Cavusoglu Colak, Mesut Ali Haliscelik, Fatma Acil, Behzat Can

**Affiliations:** 1 Department of Obstetrics and Gynecology, Division of Gynecologic Oncology, Diyarbakir Gazi Yasargil Research and Training Hospital, 21070 Diyarbakir, Turkey; erkmendnz@gmail.com (A.D.E.); drsedatakgol@gmail.com (S.A.); mesuthaliscelik@hotmail.com (M.A.H.); drbehzatcan@gmail.com (B.C.); 2 Department of Obstetrics and Gynecology, Diyarbakir Gazi Yasargil Research and Training Hospital, 21070 Diyarbakir, Turkey; dr.kubracakar@gmail.com (K.C.Y.); cvs_gul_21@hotmail.com (G.C.C.); 3 Department of Anesthesiology and Reanimation, Diyarbakir Gazi Yasargil Research and Training Hospital, 21070 Diyarbakir, Turkey; acilfatma@gmail.com

**Keywords:** vNOTES, hysterectomy, regional anesthesia, combined spinal epidural anesthesia, general anesthesia, postoperative nausea and vomiting, recovery, enhanced recovery after surgery

## Abstract

*Background and Objectives*: Vaginal natural orifice transluminal endoscopic surgery, vNOTES, has become an increasingly preferred minimally invasive option for benign hysterectomy. General anesthesia is still the routine choice, yet regional methods such as combined spinal epidural anesthesia may support a smoother postoperative course. Although the use of vNOTES is expanding, comparative information on anesthetic approaches remains limited, and its unique physiologic setting requires dedicated evaluation. To compare combined spinal epidural anesthesia with general anesthesia for benign vNOTES hysterectomy, focusing on postoperative nausea and vomiting, recovery quality, and intraoperative physiologic safety. *Materials and Methods*: This retrospective cohort study was conducted in a single center and identified women who underwent benign vNOTES hysterectomy between March 2024 and August 2025 from electronic medical records. Participants received either combined spinal epidural anesthesia or general anesthesia according to routine clinical practice. All patients were managed within an enhanced recovery pathway that incorporated standardized analgesia and prophylaxis for postoperative nausea and vomiting. The primary outcome was the incidence of postoperative nausea and vomiting during the first day after surgery. Secondary outcomes included time to discharge from the recovery unit, pain scores at set postoperative intervals, early functional recovery, patient satisfaction and physiologic parameters extracted from intraoperative monitoring records. Analyses were performed according to the anesthesia group documented in the medical files. *Results*: One hundred forty patients met inclusion criteria and were included in the analysis. Combined spinal epidural anesthesia was linked to a lower incidence of postoperative nausea and vomiting, a shorter stay in the post-anesthesia care unit, and reduced pain scores in the first 24 h (adjusted odds ratio 0.32, ninety five percent confidence interval 0.15 to 0.68). Early ambulation and oral intake were reached sooner in the combined spinal epidural group, with higher overall satisfaction also noted. Adherence to ERAS elements was similar between groups, with no meaningful differences in early feeding, mobilization, analgesia protocols or PONV prophylaxis. During the procedure, combined spinal epidural anesthesia produced more episodes of hypotension and bradycardia, while general anesthesia was linked to higher airway pressures and lower oxygen saturation. Complication rates within the first month were low in both groups. *Conclusions*: In this observational cohort study, combined spinal epidural anesthesia was associated with lower postoperative nausea, earlier recovery milestones and greater patient comfort compared with general anesthesia. Hemodynamic instability occurred more often with neuraxial anesthesia but was transient and manageable. While these findings point to potential recovery benefits for some patients, the observational nature of the study and the modest scale of the differences necessitate a cautious interpretation. They should be considered exploratory rather than definitive. The choice of anesthesia should therefore be individualized, weighing potential recovery benefits against the risk of transient hemodynamic effects. Larger and more diverse studies are needed to better define patient selection and clarify the overall risk benefit balance. These findings should be interpreted cautiously and viewed as hypothesis-generating rather than definitive evidence supporting one anesthetic strategy over another.

## 1. Introduction

Minimally invasive surgery has reshaped the management of benign gynecologic conditions. Vaginal natural orifice transluminal endoscopic surgery, vNOTES, combines the advantages of the vaginal route with the visual clarity of endoscopy, allowing less abdominal trauma and faster recovery while maintaining the precision needed for hysterectomy procedures [1,2,3,4]. As experience with this approach grows, interest has shifted toward perioperative strategies that support stable intraoperative conditions and a predictable recovery pathway.

The choice of anesthesia is central to this effort. General anesthesia remains the most common approach in laparoscopic and endoscopic gynecologic surgery and offers secure airway control with reliable surgical conditions. Even so, it is associated with postoperative nausea and vomiting, greater need for analgesia and slower return to functional activity [5,6,7,8,9]. Regional techniques, including spinal and combined spinal epidural anesthesia, have been used in selected laparoscopic procedures and have shown potential benefits such as lower rates of postoperative nausea, reduced pain and earlier ambulation [10,11,12,13,14]. Their use, however, raises concerns about hemodynamic stability, particularly during pneumoperitoneum and steep positioning, where hypotension, bradycardia and impaired ventilation may occur [15,16].

Despite a growing literature on anesthesia for laparoscopic hysterectomy, information specific to vNOTES remains limited. Most available reports focus on surgical feasibility and general perioperative safety rather than anesthetic performance or recovery quality [1,2,3,4]. Only a small number of studies have compared general anesthesia with combined spinal epidural anesthesia in this setting, and none have examined intraoperative physiology together with patient centered recovery within an enhanced recovery pathway. This gap limits the ability to guide anesthetic selection for a procedure that has its own physiologic demands.

We therefore designed a retrospective observational study to compare general anesthesia with combined spinal epidural anesthesia in women undergoing benign vNOTES hysterectomy. The main outcome was postoperative nausea and vomiting during the first day after surgery. Secondary outcomes included time to discharge from the recovery unit, pain patterns and early functional recovery. A structured set of physiologic measurements was also collected to describe hemodynamic and ventilatory responses during the procedure. Our hypothesis was that combined spinal epidural anesthesia would reduce postoperative nausea and support a smoother recovery while preserving acceptable intraoperative stability.

## 2. Materials and Methods

This retrospective observational cohort study was conducted in a single tertiary center, the Diyarbakır Gazi Yaşargil Training and Research Hospital, Department of Obstetrics and Gynecology, between March 2024 and August 2025. The study protocol was approved by the Institutional Review Board of the University of Health Sciences, Diyarbakır Gazi Yaşargil Training and Research Hospital (Approval number 496, approval date 23 May 2025). Written informed consent was obtained from all participants. The study adhered to the ethical standards of the Declaration of Helsinki [17]. All exposure, outcome and physiologic variables were extracted from routine electronic medical records; no prospective recruitment or protocol-driven data collection was performed.

Because anesthesia was selected according to routine clinical practice and patient preference, treatment allocation was not randomized This approach reflects routine clinical practice and may introduce selection bias, which was considered when interpreting the findings.

**Figure 1:**
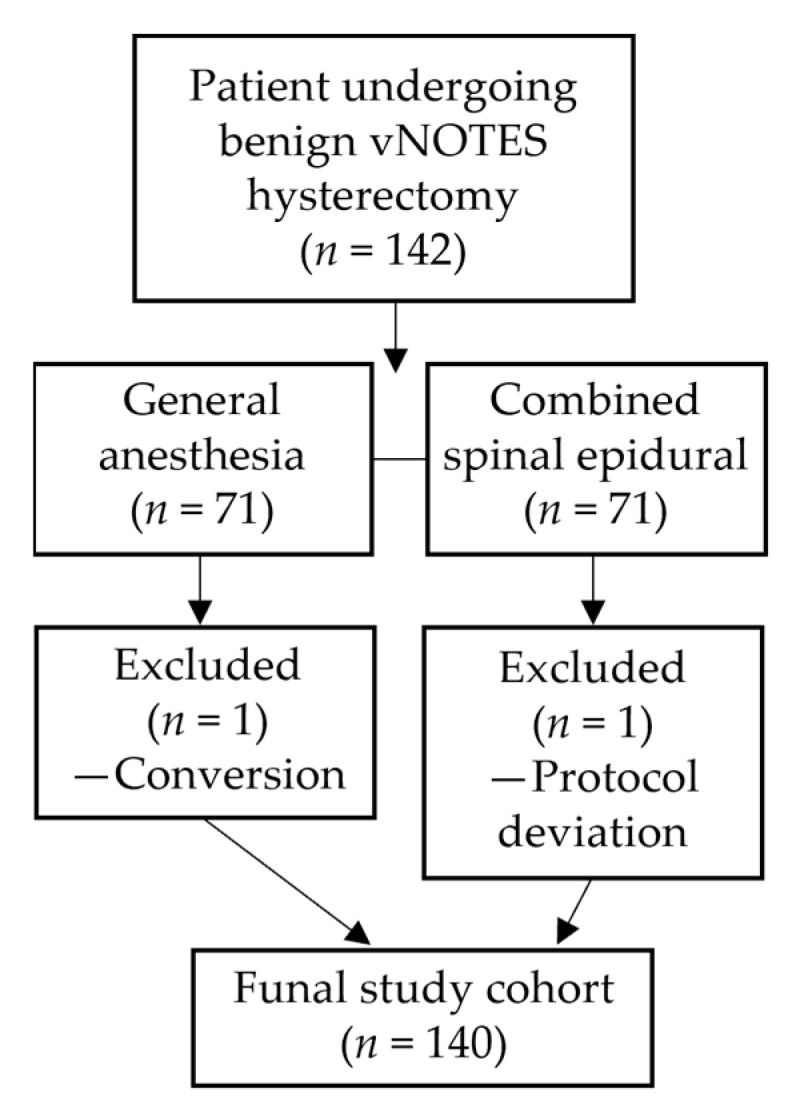
Flow of patients included in the retrospective cohort. A total of one hundred forty two women who underwent benign vNOTES hysterectomy were identified from medical records, with seventy managed with general anesthesia and seventy with combined spinal epidural anesthesia. Two patients were excluded from analysis because of conversion to open surgery or a protocol deviation. The final study cohort consisted of one hundred forty patients.

Eligible participants were women aged between eighteen and seventy five years who underwent benign vNOTES total hysterectomy with a uterine size of fourteen weeks or less and no preoperative suspicion of malignancy. Patients with American Society of Anesthesiologists physical status of one to three were included if complete perioperative records and routine clinical follow up information were available. Exclusion criteria were malignancy, emergency surgery, a body mass index of forty or higher, coagulopathy, severe valvular or unstable cardiopulmonary disease, contraindications to neuraxial anesthesia or cognitive impairment documented in the medical file. All patients undergoing benign vNOTES hysterectomy during the study period were screened for eligibility, and two patients were excluded, one because of conversion to open surgery and one due to a protocol deviation, as summarized in Figure 1.

Patients were managed with either general anesthesia or combined spinal epidural anesthesia according to routine clinical practice. The choice of anesthetic technique was determined entirely by the attending anesthesiologist in consultation with the patient and was unrelated to the conduct of the study. No study-specific criteria, matching procedures or stratified assignment methods were used to influence which patients received either technique. Baseline comparability was assessed through descriptive stratification of age, body mass index and ASA physical status, which was applied analytically rather than during clinical decision making. Anesthesia providers and surgeons were aware of the anesthesia administered, whereas staff who documented postoperative nausea, pain scores and recovery parameters were not. These postoperative outcomes were recorded as part of routine nursing documentation, and the personnel responsible for these assessments did not have access to anesthesia records at the time of clinical evaluation, resulting in effective unawareness of anesthesia type. No elements of group assignment were influenced by the study, as all anesthetic decisions preceded retrospective data extraction.

All patients followed an enhanced recovery pathway, including preoperative counseling, carbohydrate loading when appropriate, maintenance of normothermia, multimodal analgesia, early mobilization and early oral intake. Adherence to individual elements of the pathway such as early mobilization, early oral intake, use of multimodal analgesia, maintenance of normothermia and administration of prophylaxis for postoperative nausea and vomiting was extracted from perioperative checklists and is reported in Supplementary Table S3. Adherence rates were similar across the two groups.

All patients underwent routine intraoperative monitoring, including continuous electrocardiography, arterial pressure measurement (noninvasive or invasive when indicated), pulse oximetry, capnography and temperature monitoring. Intraoperative opioid administration (total fentanyl dose in micrograms) was extracted from standard anesthesia records for all patients.

In the general anesthesia group, patients were monitored according to standard perioperative guidelines, including electrocardiography, noninvasive or invasive blood pressure measurement when indicated, pulse oximetry, capnography and temperature monitoring [17,18,19,20,21]. Anesthesia was induced with an intravenous hypnotic agent, an opioid and a neuromuscular blocker, followed by tracheal intubation. Maintenance was provided with a volatile anesthetic in an oxygen and air mixture, with ventilation adjusted to maintain appropriate gas exchange. Sevoflurane was typically used for maintenance, and anesthetic depth was titrated within a clinically standard MAC range according to hemodynamic responses and surgical conditions. Hemodynamic changes such as hypotension or bradycardia were managed with vasoactive medications as required. Neuromuscular blockade was monitored clinically and with train of four assessment when indicated. Ventilation was adjusted to maintain an end tidal carbon dioxide between thirty five and forty five millimeters of mercury, with airway pressures kept within a standard protective range. Anesthetic depth was guided by hemodynamic responses and end tidal agent concentration Transient drops in oxygen saturation were corrected with short increases in inspired oxygen fraction, and no recruitment maneuvers, ventilatory support or airway adjuncts were required. Prophylaxis for postoperative nausea and vomiting was administered according to the Apfel risk score, and analgesia followed a multimodal regimen that included nonopioid agents with supplemental opioid rescue when pain intensity increased [17,22,23,24,25].

In the combined spinal epidural anesthesia group, neuraxial anesthesia was initiated in the lumbar region using a standard combined spinal epidural technique. An intrathecal local anesthetic with an opioid adjunct was administered to achieve a sensory block appropriate for the planned procedure, and an epidural catheter was placed to allow supplementation when needed. Intrathecal anesthesia consisted of a local anesthetic combined with a lipophilic opioid adjunct, as routinely administered for gynecologic neuraxial anesthesia The target sensory block level was typically between T4 and T6, which provides adequate peritoneal relaxation and visualization for vNOTES hysterectomy. Patients were positioned in lithotomy and then gradually placed into Trendelenburg to avoid rapid cephalad spread of the block; steep Trendelenburg angles were avoided until block stability was confirmed. Sedation was provided when required to maintain patient comfort. Intraoperative monitoring was the same as in the general anesthesia group, including continuous assessment of ventilation through nasal capnography. End tidal carbon dioxide and airway pressures were continuously observed to ensure adequate ventilation during spontaneous or assisted breathing. Hemodynamic changes were managed according to the same thresholds applied in the general anesthesia arm. Intraoperative records were also reviewed for symptoms potentially related to cephalad block spread, including nausea, vomiting, dyspnea, impaired ventilation or oxygen desaturation. Postoperative analgesia followed a multimodal regimen similar to that used in the general anesthesia group, with the option for epidural supplementation when indicated. The epidural catheter was removed at the end of surgery, and no postoperative epidural bolus dosing or continuous epidural infusion was used. Equipment for airway support was available at the bedside throughout the procedure [21,26]. Full anesthetic medication lists and protocol details are summarized in Supplementary Table S1.

The surgical technique was standardized for all cases. Patients were placed in the lithotomy position with Trendelenburg tilt when required. Access to the pelvis was obtained through a posterior vaginal colpotomy, after which the vNOTES platform was introduced. Carbon dioxide insufflation and endoscopic visualization were used to perform the hysterectomy with standard laparoscopic instruments. The uterus was removed through the vaginal route and the vaginal cuff was closed with delayed absorbable sutures. Additional procedures such as adnexectomy or colporrhaphy were performed when indicated. Ventilation and carbon dioxide handling were adjusted according to the anesthetic approach, with close monitoring of end tidal carbon dioxide in both groups. Procedures were performed by surgeons experienced in vNOTES, minimizing variability due to operator learning curve.

A structured intraoperative physiologic dataset was extracted from medical records after surgery. Physiologic variables were recorded by the anesthesia team during clinical care and retrieved from standardized monitoring outputs. Hemodynamic variables included arterial pressure and heart rate, with documentation of hypotension, bradycardia and the use of vasoactive medications. Hypotension was defined as a mean arterial pressure below 65 mmHg or a decrease of 20 percent or more from baseline lasting at least one minute. Bradycardia was defined as a heart rate below 50 beats per minute. An episode was counted each time these thresholds were exceeded, and duration was calculated from the onset of the abnormal value until its resolution. These definitions were applied uniformly across both groups. Respiratory variables included end tidal carbon dioxide, airway pressure and oxygen saturation. Measures of anesthetic depth, fluid administration, blood loss, urine output and core temperature were also recorded.

The primary outcome was the presence of postoperative nausea and vomiting during the first day after surgery, defined as any nausea, vomiting or rescue antiemetic use. Secondary outcomes included time to discharge from the recovery unit, postoperative pain intensity measured with a visual analogue scale at predetermined intervals, time to first ambulation, time to first oral intake, length of hospital stay, patient satisfaction assessed with a five point Likert scale and complications within thirty days graded according to the Clavien Dindo system [27,28,29,30].

All eligible cases during the study period were included in this retrospective cohort. A contextual estimate of power was calculated to assess whether the available sample size was adequate for detecting clinically meaningful differences. Prior literature reports postoperative nausea and vomiting rates of approximately forty five percent under general anesthesia and twenty percent under neuraxial techniques. Assuming this baseline difference, a two sided alpha of zero point zero five and a target power of eighty percent, a total sample of one hundred forty patients would be sufficient to detect this magnitude of effect. This estimate is provided solely to contextualize the adequacy of the dataset and does not reflect prospective sample size planning. Despite multivariable adjustment, residual confounding due to unmeasured factors cannot be excluded.

Statistical analysis was planned before the extraction of clinical data. All analyses were performed according to the anesthesia group recorded in the medical files. Continuous outcomes were compared with analysis of covariance, repeated pain measurements were examined with mixed models and categorical variables were compared with chi square or Fisher exact tests. Binary outcomes were modeled with logistic regression, count outcomes with negative binomial regression and continuous outcomes with analysis of covariance. Continuous covariates, including age, body mass index and operative time, were treated as linear terms after verifying absence of non-linearity. The selection of regression families was based on the distributional characteristics of each outcome. Non normally distributed variables were analyzed with appropriate non parametric methods Continuous variables are reported as mean and standard deviation when approximately normally distributed, and as median with interquartile range otherwise. Categorical variables are presented as counts with percentages. Missing data were handled with complete case analysis when minimal or with multiple imputation when more extensive [31,32]. Missingness was below five percent for all primary and secondary outcomes, and physiologic intraoperative variables were fully available. When imputation was required, multiple imputation with chained equations (m = 20) was performed including age, body mass index, ASA class, Apfel score, anesthesia type, operative time and all outcomes used in adjusted models, and pooled estimates were obtained using Rubin’s rules. Covariates included in the multivariable models were age, body mass index, ASA class, Apfel risk score, operative time and intraoperative opioid administration; the full covariate structure for each regression model is provided in Supplementary Table S2. Regression models were selected to account for baseline differences and to improve precision of effect estimates, as recommended for observational studies. Adjusted estimates were obtained from prespecified models that included clinically relevant covariates. All analyses were carried out using standard statistical software. All variables included in our statistical models were determined in advance, drawing on established medical knowledge and findings from earlier research. This approach ensured that our model development was grounded in clinical science, rather than being influenced by patterns observed in our specific dataset. Intraoperative opioid dose was prespecified as a covariate in multivariable models due to its known association with postoperative nausea and vomiting.

## 3. Results

### 3.1. Participant Flow and Baseline Characteristics

Between March 2024 and August 2025, one hundred forty two women underwent benign vNOTES hysterectomy in our center. Seventy were managed with general anesthesia and seventy with combined spinal epidural anesthesia. Two patients were excluded from analysis because of conversion to open surgery or a protocol deviation, resulting in a final study population of one hundred forty patients. Follow up information at thirty days was available for all patients. Missing data for postoperative outcomes were rare and did not exceed five percent for any variable. The study flow is summarized in Figure 1.

Baseline demographic and clinical characteristics were similar in the two groups (Table 1). The average age was just over fifty years in both groups and the distribution of American Society of Anesthesiologists physical status was comparable. The median Apfel score was two in each group, indicating a similar baseline risk of postoperative nausea. Other characteristics such as body mass index, hypertension, diabetes and operative time were also balanced.

### 3.2. Intraoperative Physiologic and Anesthetic Parameters

Intraoperative physiologic patterns differed between the two anesthetic approaches. Patients who received combined spinal epidural anesthesia had more episodes of hypotension and bradycardia, and these events lasted longer, leading to greater use of vasoactive medications. In contrast, general anesthesia was associated with higher airway pressures and slightly lower oxygen saturation values. Measures of end tidal carbon dioxide, blood loss, fluid balance and core temperature were similar across groups (Table 2). Intraoperative opioid administration was lower in the combined spinal epidural group compared with general anesthesia, consistent with the use of neuraxial supplementation. All intraoperative events were transient and resolved with routine measures. Episodes of oxygen desaturation under general anesthesia were corrected with brief increases in inspired oxygen fraction, and no recruitment maneuvers, ventilatory support or airway adjuncts were required. There were no documented episodes of intraoperative nausea, vomiting, dyspnea, impaired ventilation or respiratory depression in the combined spinal epidural anesthesia group. All intraoperative events were transient and were managed using standard clinical interventions. These events did not require any deviation from the planned surgical procedure, a change in the primary anesthetic technique, or an alteration to the standard postoperative care pathway.

### 3.3. Primary Outcome and Recovery Measures

Postoperative nausea and vomiting during the first day after surgery was less frequent in the combined spinal epidural anesthesia group than in the general anesthesia group. Patients who received regional anesthesia also left the recovery unit sooner. Postoperative pain followed a similar pattern, with consistently lower scores in the regional anesthesia group throughout the first day. The overall pain burden, reflected by the area under the curve, was reduced as well (Table 3).

### 3.4. Recovery and Safety Outcomes

Patients in the combined spinal epidural anesthesia group reached functional recovery milestones earlier, including ambulation and oral intake. Hospital stay tended to be shorter and patient satisfaction scores were higher in this group. Complications within thirty days were uncommon and showed no meaningful differences between the two anesthetic approaches (Table 4).

## 4. Discussion

This retrospective observational cohort study provides one of the earliest structured evaluations of combined spinal epidural anesthesia and general anesthesia in benign vNOTES hysterectomy. Combined spinal epidural anesthesia was associated with less postoperative nausea, faster recovery of basic functions and higher patient satisfaction. These advantages were accompanied by more intraoperative hypotension and bradycardia, although these events were short lived and responded well to standard vasoactive treatment. General anesthesia provided more stable hemodynamics but was linked with higher airway pressures and lower oxygen saturation during pneumoperitoneum and Trendelenburg positioning.

The observed patterns align with earlier research in laparoscopic gynecologic surgery, where regional techniques have also been correlated with reduced postoperative nausea and lower opioid requirements [5,6,7,8,9,22,23,24,25]. Differences in opioid exposure may also have contributed to the lower rates of postoperative nausea and vomiting observed in the combined spinal epidural group. The reduction in postoperative nausea in our cohort was similar to these earlier observations. The favorable pain profile observed with combined spinal epidural anesthesia is also in line with studies in laparoscopic hysterectomy and other minimally invasive procedures, where neuraxial techniques have been associated with more comfortable recovery and earlier mobilization [26,33,34,35]. These findings suggest that the benefits reported in other minimally invasive settings may extend to vNOTES, a technique with unique physiologic demands due to its use of pneumoperitoneum and pelvic positioning. However, the absolute differences in early recovery outcomes were modest, and their clinical relevance should be interpreted cautiously in light of the observational design and variation in anesthetic exposure.

The physiologic patterns observed in this study offer useful insight for anesthetic decision making in vNOTES hysterectomy. The higher incidence of hypotension and bradycardia with combined spinal epidural anesthesia is consistent with the expected effects of sympathetic blockade [33], and these events were brief and readily treated. General anesthesia, in contrast, produced higher airway pressures and occasional reductions in oxygen saturation, findings that align with earlier reports in laparoscopic surgery [12,13,14,15,16,17]. These physiologic differences may help anesthesiologists match the anesthetic technique to patient specific risks and perioperative priorities. In particular, the combination of sympathetic blockade and cephalad spread during Trendelenburg positioning may have contributed to the transient hypotension and bradycardia observed in the combined spinal epidural group. Importantly, these hemodynamic events were short in duration, responded promptly to routine vasoactive management and did not result in procedural delay or prolongation of postoperative recovery, indicating that they were clinically manageable despite their higher incidence.

The associations described here indicate that a neuraxial approach could provide specific recovery advantages in appropriately selected individuals. This choice, however, must be balanced against the more frequent, though transient, hemodynamic alterations it entailed. At the same time, general anesthesia remains appropriate for patients with limited cardiopulmonary reserve or when controlled ventilation is essential. The systematic capture of intraoperative physiologic data strengthens the applicability of these results by providing a clearer understanding of how each technique behaves under the unique demands of vNOTES. Some of the observed recovery differences may also reflect reduced intraoperative opioid exposure and lower inhalational requirements in the combined spinal epidural group, making it difficult to isolate the effect of the neuraxial component alone. Although early ambulation and oral intake occurred sooner in the combined spinal epidural group, the absolute differences were modest and may not translate into substantial improvements in overall length of stay or functional independence. The possibility that epidural supplementation contributed to the improved pain outcomes observed in this study should also be acknowledged. Observed differences likely reflect differences in overall anesthetic exposure rather than the neuraxial component alone. Ultimately, because each strategy functions as an integrated clinical bundle, the outcomes we observed are best understood as the net effect of a coordinated set of physiological and pharmacological changes. The primary contribution of this work lies in applying existing anesthetic principles to the unique vNOTES environment and in proposing a physiology-based rationale to guide future research on patient selection.

Strengths of this study include its structured design, the use of a standardized enhanced recovery pathway, a sample size calculation based on clinically relevant outcomes and high follow up completeness with blinded assessment of postoperative outcomes. This analysis is subject to important limitations stemming from its single-center, retrospective nature. The non-randomized assignment of anesthetic technique, based on standard clinical decisions and patient-specific factors, introduces a potential for selection bias and confounding that statistical models cannot entirely resolve. Consequently, the relationships identified here should be considered preliminary and are intended to inform the design of more rigorous, prospective studies, not to establish definitive causal links. The purpose of this analysis is not to recommend a new practice, but rather to contribute nuanced data that may assist in individualized clinical judgment and to identify specific questions, particularly regarding which patients might benefit most, for future, more targeted research.

In particular, reduced exposure to intraoperative opioids and volatile anesthetic agents in the combined spinal epidural group may have contributed to some of the observed recovery differences, limiting the ability to attribute these associations solely to the neuraxial technique. The physiologic dataset did not include biochemical markers of surgical stress, and outcomes beyond thirty days were not assessed. These factors should be taken into account when interpreting the generalizability of the findings. Although statistically significant, some observed differences were modest in magnitude and may not translate into clinically meaningful benefits for all patients.

Future studies conducted in larger and more diverse centers would help confirm the generalizability of these findings. The inclusion of patient reported outcome measures may offer a more complete view of recovery from the patient’s perspective. Further work could also explore blended anesthetic approaches that aim to balance hemodynamic stability with the recovery benefits observed in regional techniques.

## 5. Conclusions

In this retrospective cohort study, combined spinal epidural anesthesia was associated with lower rates of postoperative nausea, earlier attainment of early recovery milestones, and greater patient-reported comfort compared with general anesthesia in patients undergoing benign vNOTES hysterectomy. Neuraxial anesthesia was accompanied by a higher incidence of transient intraoperative hemodynamic instability, which was readily manageable, whereas general anesthesia afforded more stable ventilatory control. These findings suggest potential recovery-related advantages of combined spinal epidural anesthesia in selected patients; however, the modest absolute effect sizes and the non-randomized observational design necessitate cautious interpretation. Accordingly, anesthetic selection should be individualized, balancing potential recovery benefits against patient-specific risks and perioperative priorities. These findings should be interpreted cautiously and viewed as hypothesis-generating rather than definitive evidence supporting one anesthetic strategy over another.

## Figures and Tables

**Table 1:** Baseline demographic and clinical characteristics of the study population.

Variable	GA (*n* = 70)	CSEA (*n* = 70)	*p*-Value
Age (years), mean ± SD	52.3 ± 9.8	51.7 ± 10.1	0.72
BMI (kg/m^2^), mean ± SD	28.4 ± 3.5	28.6 ± 3.2	0.72
ASA class III, *n* (%)	10 (14.3%)	11 (15.7%)	1.00
Apfel score, median [IQR]	2 [2,3]	2 [2,3]	1.00
Hypertension, *n* (%)	19 (27.1%)	20 (28.6%)	1.00
Diabetes mellitus, *n* (%)	8 (11.4%)	7 (10.0%)	1.00
Operative time (min), mean ± SD	98.2 ± 16.5	97.5 ± 15.9	0.80

Values are presented as mean and standard deviation for approximately normally distributed variables, as median with interquartile range for non-parametric variables and as number with percentage for categorical variables. *p*-values were calculated using two sample *t*-tests for continuous variables, chi square tests for categorical variables and Mann–Whitney U tests for non parametric variables. ASA, American Society of Anesthesiologists; BMI, body mass index; GA, general anesthesia; CSEA, combined spinal epidural anesthesia; IQR, interquartile range.

**Table 2:** Intraoperative physiologic parameters comparing general anesthesia and combined spinal epidural anesthesia.

Outcome	General Anesthesia (*n* = 70)	Combined Spinal Epidural Anesthesia (*n* = 70)	Adjusted Effect	95 Percent Confidence Interval	*p* Value
Hypotension episodes, median and interquartile range	1 (0 to 2)	3 (2 to 4)	Adjusted rate ratio 2.9	1.9 to 4.3	<0.001
Duration of hypotension (minutes), mean ± SD	6.1 ± 5.4	14.3 ± 9.2	Adjusted mean difference 8.2	6.0 to 10.5	<0.001
Bradycardia, *n* (%)	3 (4.3)	11 (15.7)	Adjusted odds ratio 4.1	1.1 to 15.3	0.03
Phenylephrine use (micrograms), mean ± SD	90 ± 55	180 ± 75	Adjusted mean difference 90	65 to 115	<0.001
End tidal carbon dioxide (mmHg), mean ± SD	37.5 ± 3.8	38.2 ± 4.2	Adjusted mean difference 0.7	−0.6 to 2.0	0.28
Peak airway pressure (cm H_2_O), mean ± SD	22.4 ± 3.6	17.1 ± 2.8	Adjusted mean difference −5.3	−6.2 to −4.4	<0.001
Lowest oxygen saturation (%), mean ± SD	93.1 ± 2.8	95.2 ± 1.9	Adjusted mean difference 2.1	1.2 to 2.9	0.004
Intravenous fluids (mL), mean ± SD	1100 ± 250	1080 ± 260	Adjusted mean difference −20	−95 to 55	0.61
Estimated blood loss (mL), mean ± SD	120 ± 40	115 ± 38	Adjusted mean difference −5	−20 to 10	0.48
Urine output (mL), mean ± SD	180 ± 60	175 ± 55	Adjusted mean difference −5	−25 to 15	0.65
Lowest core temperature (°C), mean ± SD	36.0 ± 0.4	36.1 ± 0.5	Adjusted mean difference 0.1	−0.1 to 0.2	0.27

Values are presented as mean and standard deviation for approximately normally distributed variables, as median with interquartile range for non-parametric variables and as number with percentage for categorical variables. Adjusted effects are reported as adjusted rate ratios, adjusted odds ratios or adjusted mean differences.

**Table 3:** Primary and early recovery outcomes in patients receiving general anesthesia or combined spinal epidural anesthesia.

Outcome	General Anesthesia (*n* = 70)	Combined Spinal Epidural Anesthesia (*n* = 70)	Adjusted Effect	95 Percent Confidence Interval	*p* Value
Postoperative nausea and vomiting, *n* (%)	32 (45.7)	15 (21.4)	Adjusted odds ratio 0.32	0.15 to 0.68	0.012
Time to discharge from recovery unit (minutes), mean ± SD	92 ± 15	76 ± 14	Adjusted mean difference −16	−20 to −12	0.004
Pain score at hour 1, mean ± SD	4.8 ± 1.2	3.2 ± 1.1	Adjusted mean difference −1.6	−2.0 to −1.2	0.001
Pain score at hour 6, mean ± SD	3.9 ± 1.0	2.5 ± 0.9	Adjusted mean difference −1.4	−1.7 to −1.0	<0.001
Pain score at hour 24, mean ± SD	2.8 ± 0.8	1.7 ± 0.7	Adjusted mean difference −1.1	−1.4 to −0.8	0.002
Pain burden, area under the curve (0 to 24 h), mean ± SD	67 ± 12	43 ± 10	Adjusted mean difference −24	−28 to −20	<0.001

Values are presented as mean and standard deviation for approximately normally distributed variables, as number with percentage for categorical variables and as adjusted mean differences for covariate adjusted continuous outcomes. Adjusted effects are derived from multivariable logistic regression for binary outcomes and analysis of covariance for continuous outcomes.

**Table 4:** Early postoperative recovery outcomes and thirty-day safety events.

Outcome	General Anesthesia (*n* = 70)	Combined Spinal Epidural Anesthesia (*n* = 70)	Effect Estimate	95 Percent Confidence Interval	*p* Value
Time to ambulation (hours), mean ± SD	11.2 ± 3.0	7.6 ± 2.4	Adjusted mean difference −3.6	−4.4 to −2.8	<0.001
Time to oral intake (hours), mean ± SD	15.8 ± 4.2	11.9 ± 3.5	Adjusted mean difference −3.9	−5.1 to −2.7	0.001
Length of hospital stay (days), mean ± SD	2.9 ± 0.8	2.5 ± 0.7	Adjusted mean difference −0.4	−0.6 to −0.2	0.021
Patient satisfaction, median and interquartile range	4 (3 to 4)	5 (4 to 5)	Adjusted odds ratio 2.6	1.4 to 4.8	0.017
Complications within thirty days, *n* (%)	3 (4.3)	2 (2.9)	Odds ratio 0.67	0.11 to 3.95	0.64

Values for continuous early recovery outcomes are presented as mean and standard deviation, and categorical outcomes are presented as number with percentage. Thirty day safety events are reported as number with percentage. Early recovery outcomes were available for all patients, and thirty day safety events were documented for all patients.

## Data Availability

The datasets used and/or analyzed during the current study are available within the manuscript or from the corresponding author upon reasonable request.

## Supplementary Materials

The following supporting information can be downloaded at: https://www.mdpi.com/article/10.3390/medicina62010154/s1, Table S1: Summary of anesthetic medications, intraoperative protocols and monitoring parameters used in the general anesthesia and combined spinal epidural anesthesia groups; Table S2: Covariates included in each multivariable regression model, including logistic regression for binary outcomes, negative binomial regression for count outcomes and analysis of covariance for continuous outcomes; Table S3: Adherence to key ERAS components, including early mobilization, early oral intake, normothermia maintenance, multimodal analgesia and standardized prophylaxis for postoperative nausea and vomiting.

## Abbreviations

ASA	American Society of Anesthesiologists
BMI	Body mass index
CSEA	Combined spinal-epidural anesthesia
ERAS	Enhanced Recovery After Surgery
GA	General anesthesia
IQR	Interquartile range
MAC	Minimum alveolar concentration
PONV	Postoperative nausea and vomiting
SD	Standard deviation
vNOTES	Vaginal natural orifice transluminal endoscopic surgery
