# Supplementary material for: Does Regional Anesthesia Improve Recovery After vNOTES Hysterectomy? A Comparative Observational Study
Source: Medicina (Kaunas). 2026 Jan 13;62(1):154. doi: 10.3390/medicina62010154 (PMC12843978; doi:10.3390/medicina62010154)
Supplement: Supplementary file 1 [file medicina-62-00154-s001.zip › medicina-4066195-supplementary.pdf]

Supplementary Materials

Supplementary Table S1. Summary of anesthetic medications and perioperative management protocols used in the study

Monitoring	Standard perioperative monitoring including electrocardiography, arterial pressure (noninvasive or invasive when indicated), pulse oximetry, capnography and temperature monitoring.	Same monitoring as general anesthesia, with nasal capnography for spontaneously breathing patients.
Induction	Intravenous hypnotic agent, opioid and neuromuscular blocker followed by tracheal intubation.	Intrathecal administration of local anesthetic with an opioid adjunct, followed by epidural catheter placement for supplementation. Sedation provided when required.
Maintenance / Intraoperative Management	Volatile anesthetic in oxygen and air mixture. Ventilation adjusted to maintain normocapnia. Hemodynamic changes treated with vasoactive agents according to clinical need.	Sensory block maintained with epidural supplementation when indicated. Ventilation and carbon dioxide handling adjusted for spontaneous or assisted breathing. Hemodynamic thresholds managed with the same criteria as in general anesthesia.
Analgesia	Multimodal analgesia with nonopioid medications and opioid rescue doses as needed.	Same multimodal regimen with the option of epidural supplementation for analgesia.
PONV Prophylaxis	Administered according to Apfel risk score using single, dual or triple therapy.	Same protocol as general anesthesia.
Airway Management	Tracheal intubation for all patients.	Airway rescue equipment available throughout the procedure.
Additional Notes	Ventilation tailored to laparoscopic conditions as needed.	Block level monitored clinically; adjustments made via epidural catheter.

This table provides an overview of the anesthetic medications and perioperative management practices applied in the general anesthesia and combined spinal epidural anesthesia groups. The summary includes monitoring approaches, induction and maintenance strategies, postoperative analgesia, prophylaxis for postoperative nausea and vomiting and airway management precautions. The table is intended to complement the Methods section by outlining protocol components that support reproducibility while keeping the main text focused on the comparative clinical outcomes.

Supplementary Table S2. Covariates included in each multivariable regression model.

Outcome	Model type	Covariates included
Postoperative nausea and vomiting (binary)	Logistic regression	Age; body mass index; ASA physical status; Apfel risk score; operative time; intraoperative opioid administration
Time to discharge from recovery unit (continuous)	Analysis of covariance (ANCOVA)	Age; body mass index; ASA physical status; Apfel risk score; operative time; intraoperative opioid administration
Pain scores at 1, 6 and 24 hours (repeated continuous outcomes)	Mixed-effects linear model with random intercept	Age; body mass index; ASA physical status; Apfel risk score; operative time; intraoperative opioid administration; postoperative time point
Pain burden (AUC 0–24 hours)	Analysis of covariance (ANCOVA)	Age; body mass index; ASA physical status; Apfel risk score; operative time; intraoperative opioid administration

Hypotension episodes (count)	Negative binomial regression	Age; body mass index; ASA physical status; Apfel risk score; operative time; intraoperative opioid administration
Duration of hypotension (continuous)	Analysis of covariance (ANCOVA)	Age; body mass index; ASA physical status; Apfel risk score; operative time; intraoperative opioid administration
Bradycardia (binary)	Logistic regression	Age; body mass index; ASA physical status; Apfel risk score; operative time; intraoperative opioid administration
Peak airway pressure (continuous)	Analysis of covariance (ANCOVA)	Age; body mass index; ASA physical status; Apfel risk score; operative time; intraoperative opioid administration
Lowest oxygen saturation (continuous)	Analysis of covariance (ANCOVA)	Age; body mass index; ASA physical status; Apfel risk score; operative time; intraoperative opioid administration

Covariates included in each adjusted regression model. Negative binomial regression was used for count outcomes; logistic regression for binary outcomes; and analysis of covariance for continuous outcomes. Mixed-effects linear models were applied to repeated postoperative pain scores. Covariate selection was based on clinical relevance and existing literature regarding postoperative nausea, recovery dynamics and intraoperative physiology. All variables included in imputation models are listed in Table S2.

Supplementary Table S3. Adherence to key ERAS components in the general anesthesia and combined spinal epidural anesthesia groups.

ERAS Component	General Anesthesia (n = 70)	Combined Spinal Epidural Anesthesia (n = 70)
Early mobilization (within 12 h)	67 (95.7%)	68 (97.1%)
Early oral intake (within 6 h)	66 (94.3%)	67 (95.7%)
Maintenance of normothermia	69 (98.6%)	69 (98.6%)
Multimodal analgesia applied	70 (100%)	70 (100%)
PONV prophylaxis per Apfel score	70 (100%)	70 (100%)

Adherence to individual ERAS components was extracted from perioperative checklists. No meaningful differences were observed between groups, and overall adherence exceeded 94 percent for all key components.
